# Introducing Virtual Visit Blocks to Optimize Space in Primary Care Practice

**DOI:** 10.1089/tmr.2025.0018

**Published:** 2025-05-07

**Authors:** Susan Pohl, Lindsey Garcia, Sofia Loucao, Erin McCormack, Jennifer Vogt, Bernadette Kiraly

**Affiliations:** University of Utah, Salt Lake City, Utah, USA.

**Keywords:** health care workforce, primary care, telemedicine, virtual

## Abstract

The COVID-19 pandemic significantly accelerated the adoption of telehealth in primary care settings, with many health care systems planning to continue offering virtual care indefinitely. This brief report describes the implementation of virtual visit (VV) blocks to optimize telemedicine visits and expand clinic workforce capacity. VV blocks, dedicated time slots exclusively for telemedicine, were introduced to free up physical space for additional on-site providers. By pairing the introduction of VV blocks with new provider hires, our health system successfully expanded its workforce, increasing provider full-time equivalents in our pilot clinic from 8.51 to 10.25. These changes led to improved access, higher visit volumes, and similar patient satisfaction. Providers also reported benefits in terms of work-life balance and efficiency. The VV block model proved effective in addressing space and resource constraints, improving both operational outcomes and financial sustainability. The success of this pilot was replicated in a second clinic, demonstrating scalability. The long-term viability of telehealth initiatives hinges on the continuation of insurance payment parity and legislative support for telehealth policies. This article provides insights into how telehealth integration can optimize primary care delivery while navigating operational and financial challenges.

## Background

The Covid-19 pandemic abruptly launched many health care systems into the world of virtual care/telehealth. There are many clinical care visits in primary care that are appropriate for telehealth,^1,2^ and most medical practices that introduced virtual care plan to continue to offer this care indefinitely.^3^ Telemedicine visits allow patients to receive care with no travel time, reducing needs for childcare, fuel expenses, and lost wages. These are hidden health care costs that most heavily burden lower-income patients. Telemedicine visits also provide flexibility in the delivery of medical care to patients and caregivers with mobility issues, lack of transportation, and long travel distances.^4^ There are many models for integrating telehealth visits into traditional clinical practices. A thorough literature review of Pubmed, Embase, and CINAHL was done with the aid of a medical librarian looking for telemedicine and virtual visit provider schedule optimization. There are published reports showing innovative scheduling of mental health providers based on need,^5^ intensive care unit schedule optimization via telehealth coverage,^6,7^ and emergency department provider schedule optimization using a telemedicine service hub model.^8^ The review did not reveal any reports describing schedule optimization of outpatient telemedicine visits in the primary care setting. This report describes using virtual visit scheduling blocks to optimize telehealth visits in a primary care clinic and allow expansion of the clinic workforce. In this article, we will describe the implementation and impact of virtual visit blocks and offer insights into how this approach can help practices navigate resource and space constraints.

## Methods

In 2021, most primary care providers at the University of Utah had telemedicine visits interspersed throughout their daily clinic schedule without restrictions. Through an iterative process between 2021 and 2022, several primary care physicians grouped telemedicine visits into a designated session in their schedule (or block of time), which were termed virtual visit (VV) blocks. These blocks were restricted to only allow for the scheduling of telehealth visits. The blocks allowed providers to conduct patient care remotely, freeing up clinic exam room space. In 2022, the VV block pilot was initiated to expand VV blocks thoughtfully and pair this expansion with hiring since VV blocks created clinical space for an additional provider onsite.

**FIG. 1.**
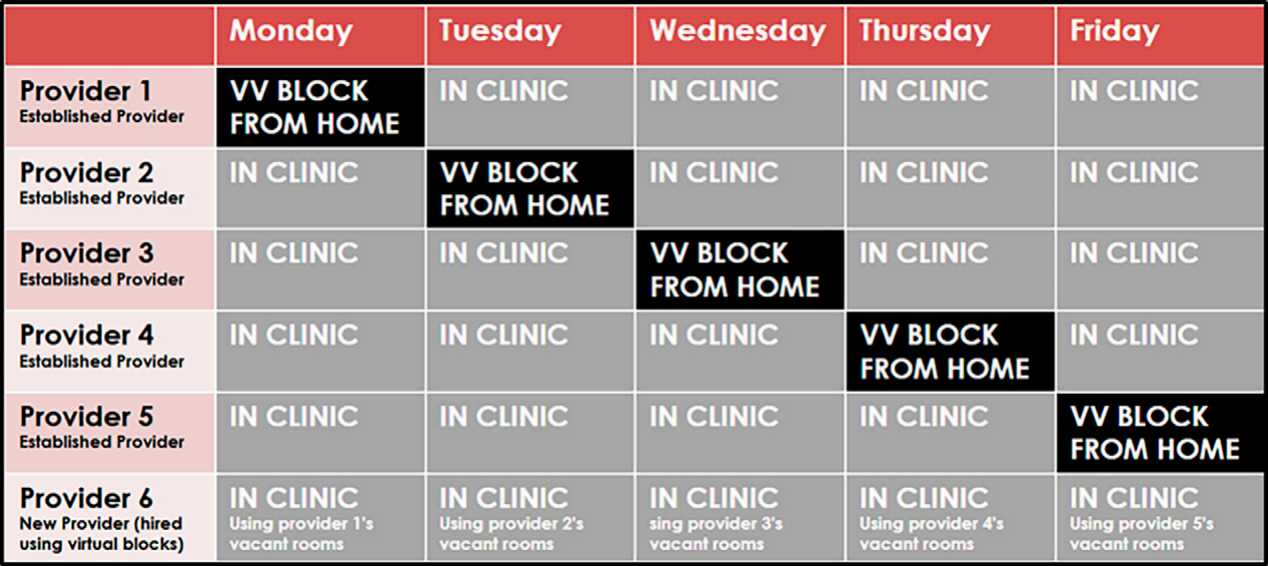
Virtual Visit (VV) Blocks Methodology. VV = virtual visit.

To begin the VV pilot, five existing primary care physicians (family medicine and internal medicine/pediatrics) in one busy clinic were approached with the pilot by clinic administration and the project management team as a way to expand the provider workforce beyond the limits of the physical clinic space. The physicians volunteered to participate in the program, and time spent in the VV blocks was included in their required full-time equivalent (FTE) hours. To begin the process of implementing VV blocks, participating providers added restrictions to their schedule 1 day a week that only allowed telemedicine visits to be scheduled and barred any in-person visits. Providers then worked remotely during VV blocks, and this created clinic space available for additional providers. Clinic administration configured the VV blocks to allow for expansion of the provider workforce. Figure 1 shows how the coordinated use of VV blocks effectively created enough space to allow expansion of the provider workforce. In this model, 5 full-time providers (providers 1 through 5 in the Fig. below) added one VV block during the week. The VV blocks and remotely working providers created space for an additional full-time provider, provider 6, to utilize the now vacant exam rooms.

Performance measures for the VV blocks before and after the additional hires included change in total visit volume, new patient visits, and patient experience regarding appointment availability. We evaluated percent change (95% confidence intervals [CI]) from pre- to post-hiring using the chi-square test for total number of visits and new patient visits. Patient experience was captured on a Likert scale (0 to 100) with five ordinal categories (0 = very poor; 25 = poor; 50 = fair; 75 = good, and 100 = very good). We evaluated patient experience from pre- to post- hiring using both the chi-square and the Wilcoxon-Mann–Whitney test for the ordinal 5 categories

## Results

Prior to the initiation of the VV pilot and subsequent hiring, the pilot clinic had a total provider clinical FTE of 8.51. There were some existing blocks for virtual care, but visits in these blocks were only 0.82% of visits. After the VV block pilot and subsequent hiring, the total provider workforce increased to 10.25 FTE, and 7% of visits occurred during VV blocks. As expected during this workforce expansion, total visits increased in the 12 months post-hiring (12.88% increase [95% CI: 11.38, 14.29], *p* = < 0.001), and new patient visits increased (3.39% increase [95% CI: −0.92, 7.70], *p* = 0.12) (Table 1). Revenue increased with workforce expansion. All patients were surveyed about their experience with scheduling and appointment availability using our standard institutional process. We found no difference in median patient experience in the general area of “ease of scheduling” before and after the additional hires (median = 100, IQR = 75 to 100 for both time periods), Wilcoxon-Mann–Whitney *p* = 0.99. The vast majority of scores before and after hiring were “100 = very good,” with a slight increase in number of perfect scores before versus after the subsequent hiring (63.7% to 64.3%, 5 category chi-square *p*-value = 0.19).

**Table 1.** Performance Measures for Virtual Blocks

	Pre-hire 12/01/2021–11/30/2022	Post-hire 12/01/2022–11/30/2023	Percent change 95% CI	*p*-value^a^
Visit Volume				
*n* (%)	40,811 (47%)	46,069 (53%)	12.88 (11.38, 14.39)	<0.001
New Patient Visits				
*n* (%)	4,339	4,486	3.39 (−0.92, 7.70)	0.12
Patient Experience^b^ regarding appointment availability				
median (25%, 75%)	100 (75, 100)	100 (75, 100)	N/A	0.99
*n* (%)			N/A	0.19
Very poor	19 (1.0)	32 (1.4)		
Poor	40 (2.1)	63 (2.8)		
Fair	175 (9.0)	197 (8.9)		
Good	475 (24.4)	499 (22.5)		
Very good	1242 (63.7)	1424 (64.3)		

^a^ Chi-square test for independent (non-paired) samples for visit volume and new patient visits. Wilcoxon-Mann–Whitney and chi-square test for patient experience.

^b^ Patient experience, *n* = 4166 (*n* = 1951 pre-hire; and *n* = 2215 post-hire), scale 1 to 100 with 0 = very poor; 25 = poor; 50 = fair; 75 = good, and 100 = very good.

## Discussion

In addition to an increase in visit volume, higher revenue, and a similar patient experience, providers gave positive feedback about the VV block structure. One primary care physician in the VV block pilot shared the following in an anonymous survey: “Virtual visits have helped my practice in multiple ways. I find virtual visits to be much more time-efficient and therefore allow for quality patient care but also better work-life balance, as I find myself able to complete visits and notes faster and be done with clinic faster. Simply not driving to and from work also gives me more time in my day.” After the successful expansion of the workforce at the first pilot site, the process was repeated at additional clinics, and similar results were achieved. The virtual block model has met the financial needs of our organization, and the model continues to be used where implemented.

### Challenges and considerations

To ensure that VV blocks met the needs of a practice, the following issues were considered:

- **Provider panel size, composition, and commitment to the VV block:** Providers with larger, established practices had more success filling the VV blocks and were given priority for inclusion in the pilot. Providers promoted telemedicine visits to their patients that were particularly suited to virtual care, like mood disorder treatment, attention and hyperactivity disorder treatment, and hormone replacement therapy. Providers volunteered to add VV blocks to their schedule and voiced commitment to the success of their VV blocks. We hired additional providers using the clinic space opened by VV blocks. VV block providers were aware that the success of the project required a long-term commitment to the VV block model since their original space for in-person care was utilized by new providers hired to work in the clinics.
- **Provider technical skills:** Providers with VV blocks completed VV blocks off-site and relied on remote technical support. Providers were comfortable with the technology and trained in contingencies when the technology was not functioning properly.
- **Diverse scheduling technology used for virtual visits:** All scheduling platforms, such as phone calls, electronic health record portals, and web-based scheduling, were available to patients seeking scheduling for in-person and virtual visits. We found it essential that all scheduling modalities facilitated this patient choice for telemedicine visits.

## Conclusion

Telemedicine visits are now an important part of primary care delivery, and when used strategically, they may be able to address space and resource constraints, patient access issues, and financial sustainability. The VV blocks described in this article represent one way to optimize scheduling of telemedicine visits in a group practice, leading to improved patient and provider experience with positive operational outcomes in both access and revenue.

The long-term sustainability of telemedicine depends on continued insurance payment parity and adoption of permanent statutory telehealth waivers by Centers for Medicare and Medicaid Services and the Drug Enforcement Administration. On March 15, 2025, the Full-Year Continuing Appropriations and Extensions Act, 2025, was passed by Congress and includes a six-month extension of telehealth flexibilities that were set to expire on March 31, 2025, now extended through September 30, 2025.^9^ These temporary provisions ensure uninterrupted access to telehealth services and give health care providers continued flexibility as Congress works toward a long-term telehealth policy. There is a risk of a virtual care “cliff” with an abrupt reversal of the improvements of access to care supported by virtual visits if these flexibilities are reversed or allowed to expire.

Despite the uncertainty due to lack of permanent congressional support, we and many other organizations have moved ahead with developing robust virtual care programs.

## Abbreviations Used

CI: confidence intervals
FTE: full-time equivalent
VV: virtual visit

## References

[1] Segal JB, Dukhanin V, Davis S. Telemedicine in primary care: Qualitative work towards a framework for appropriate use. J Am Board Fam Med 2022;35(3):507–516; doi: 10.3122/jabfm.2022.03.210229 35641038

[2] Bazzano AN, Patel T, Nauman E, et al. Optimizing telehealth for diabetes management in the Deep South of the United States: Qualitative study of barriers and facilitators on the patient and clinician journey. J Med Internet Res 2024;26:e43583; doi: 10.2196/43583 37976468 PMC10790202

[3] Ho TF, Fortenberry KT, Gardner E, et al. Perceived impact of virtual visits on access to care in family medicine during the COVID-19 pandemic: A qualitative study of benefits and challenges. J Prim Care Community Health 2023;14:21501319231220118; doi: 10.1177/21501319231220118 38140819 PMC10748621

[4] Kidholm K, Jensen LK, Johansson M, et al. Telemedicine and the assessment of clinician time: A scoping review. Int J Technol Assess Health Care 2023;40(1):e3; doi: 10.1017/S0266462323002830 38099431 PMC10859839

[5] Palmer A, Johns G, Ahuja A, et al. Optimizing an adolescent hybrid telemedical mental health service through staff scheduling using mathematical programming: Model development study. JMIR Form Res 2023;7:e43222; doi: 10.2196/43222 36976622 PMC10131707

[6] Udeh BL, Thompson NR, Honomichl RD, et al. The case for telemedicine-enhanced nighttime staffing in a Neuro-ICU. Crit Care Explor 2025;7(3):3 e1231; doi: 10.1097/CCE.0000000000001231 PMC11884836 40042218

[7] Li X, Liu D, Yang M, et al. Staffing decisions in a tele-ICU: Dedicated versus flexible resources. IISE Transactions on Healthcare Systems Engineering 2020;10(3):172–183; doi: 10.1080/24725579.2020.1749911

[8] Olanrewaju OG, Erkoc M. Physician scheduling for emergency telemedicine across multiple facilities. IISE Transactions on Healthcare Systems Engineering 2023;13(3):182–197; doi: 10.1080/24725579.2023.2201481

[9] United States. Full-Year Continuing Appropriations and Extensions Act, 2025, Pub. L. No. 119-4. U.S. Congress: Washington (DC); 2025. [cited 2025 Apr 14]. Available from: https://www.congress.gov/bill/119th-congress/house-bill/1968

